# Effects of unaltered and bioconverted mulberry leaf extracts on cellular glucose uptake and antidiabetic action in animals

**DOI:** 10.1186/s12906-019-2460-5

**Published:** 2019-03-06

**Authors:** Sang-Hyuk Jung, Joo-Hui Han, Hyun-Soo Park, Do-Hyung Lee, Seok Jin Kim, Hyun So Cho, Jong Seong Kang, Chang-Seon Myung

**Affiliations:** 1 Department of Pharmacology, Chungnam National University College of Pharmacy, 99 Daehak-ro (St.), Yuseong-gu, Daejeon, 34134 Republic of Korea; 2 MSC Annexed Food Technology Research Institute, Yangsan-si, Gyeongsangnam-do 50518 Republic of Korea

**Keywords:** Bioconversion, Insulin resistance, Insulin sensitivity, Mulberry leaf extract, Diabetic animals

## Abstract

**Background:**

Mulberry is a Korean medicinal herb that shows effective prevention and treatment of obesity and diabetes. Bioconversion is the process of producing active ingredients from natural products using microorganisms or enzymes.

**Methods:**

In this study, we prepared bioconverted mulberry leaf extract (BMLE) with Viscozyme L, which we tested in insulin-sensitive cells (i.e., skeletal muscle cells and adipocytes) and insulin-secreting pancreatic β-cells, as well as obese diabetic mice induced by co-administration of streptozotocin (100 mg/kg, IP) and nicotinamide (240 mg/kg, IP) and feeding high-fat diet, as compared to unaltered mulberry leaf extract (MLE).

**Results:**

BMLE increased the glucose uptake in C2C12 myotubes and 3 T3-L1 adipocytes and increased glucose-stimulated insulin secretion in HIT-T15 pancreatic β-cells. The fasting blood glucose levels in diabetic mice treated with BMLE or MLE (300 and 600 mg/kg, PO, 7 weeks) were significantly lower than those of the vehicle-treated group. At the same concentration, BMLE-treated mice showed better glucose tolerance than MLE-treated mice. Moreover, the blood concentration of glycated hemoglobin (HbA_1C_) in mice treated with BMLE was lower than that in the MLE group at the same concentration. Plasma insulin levels in mice treated with BMLE or MLE tended to increase compared to the vehicle-treated group. Treatment with BMLE yielded significant improvements in insulin resistance and insulin sensitivity.

**Conclusion:**

These results indicate that in the management of diabetic condition, BMLE is superior to unaltered MLE due to at least, in part, high concentrations of maker compounds (trans-caffeic acid and syringaldehyde) in BMLE.

**Electronic supplementary material:**

The online version of this article (10.1186/s12906-019-2460-5) contains supplementary material, which is available to authorized users.

## Background

Mulberry has origins in Korea, China, and Japan, and was traditionally cultivated for medicinal materials and silkworm food from all plant parts (e.g., leaves, fruit, and root bark) [1, 2]. In China and Korea, it has long been used as a treatment for obesity and diabetes. Recent studies have reported several pharmacological effects of mulberry leaves, including blood pressure stabilization [3], cholesterol reduction [4], thrombolysis [5], antioxidation [6], and antiobesity [7–9]. In particular, a number of human [10, 11] or animals [12–16] studies have reported to confirm the antidiabetic activity of mulberry extract by regulating blood glucose level.

Bioconversion is a technique used to induce the production of physiologically active ingredients by changing the structure of chemicals in natural products using biological methods such as microbial fermentation and enzyme treatment [17, 18]. Several studies have reported that bioconversion can improve the biological activity of extracts. For example, bioconverted *Citrus unshiu* peel extract with cytolase showed the ability to inhibit lipogenesis in 3 T3-L1 cells [19], and fermented soybean alleviated type 2 diabetes [20]. Moreover, bioconverted *Morinda citrifolia* (Noni) using c*heonggukjang* improved the management of type 2 diabetes [21], and fermented red ginseng by *Lactobacillus plantarum* showed the ability to regulate postprandial blood glucose levels in type 2 diabetic patients [22]. Similarly, chickpea (*Cicer arietinum* L.) bioconverted by *Rhizopus oligosporus* showed improvements in blood glucose levels [23]. It has been reported that Viscozyme L, a carbohydrate-hydrolyzing enzyme from Aspergillus aculeatus with 100 fungal β-glucanase units, was used for bioconverstion of unripe apples and this had the strongest effect on polyphenols extraction to yield components [24]. Thus, this enzyme may be a good tool for bioconversion of natural products.

Type 2 diabetes can be induced in animal models by co-administration of streptozotocin (STZ) and nicotinamide (NA) [25–29]. Although STZ destroys pancreatic β-cells and NA can partially protect β-cells, levels of N1-methylnicotinamide, a metabolite of NA, increase in the plasma when excess NA is administered, leading to oxidative stress, insulin resistance, and ultimately type 2 diabetes [30]. In type 2 diabetic animal models, glucose and glycated hemoglobin (HbA_1C_) blood levels and the ability of pancreatic β-cells to secret insulin are important indices for the evaluation of diabetes [31, 32].

In this study, we prepared bioconverted mulberry leaf extract (BMLE) using Viscozyme L, a multi-enzyme complex with a strong pectolytic activity and a wide range of carbohydrases including arabanase, cellulase, β-glucanase, hemicellulase, and xylanase (Manufacturer’s Application sheet). The ability of BMLE to uptake glucose into insulin-sensitive cells (C2C12 myotubes and 3 T3-L1 adipocytes) and secret insulin from HIT-T15 pancreatic β-cells, as well as to lower blood glucose and HbA_1C_ levels and secret insulin in obese diabetic mice induced by co-administration of STZ and NA and feeding high-fat diet, as compared to unaltered mulberry leaf extract (MLE).

## Methods

### Reagents

Dulbecco’s Modified Eagle Medium (DMEM), fetal bovine serum (FBS), RPMI-1640 medium, phosphate-buffered saline (PBS), and trypsin-EDTA were obtained from Gibco BRL (Grand Island, NE, USA). Penicillin/streptomycin was obtained from Thermo Fisher Scientific (Rockford, IL, USA). Insulin, NA, STZ, glimepiride, metformin, D-(+)-glucose, 2-deoxy-D-glucose, 3-isobutyl-1-methylxanthine (IBMX), 4-(2-hydroxyethyl)-1-piperazineethanesulfonic acid (HEPES), NaCl, KCl, MgSO_4_, CaCl_2_, KH_2_PO_4_, and NaHCO_3_ were obtained from Sigma-Aldrich (St. Louis, MO, USA). 3-(4,5-Dimethylthiazol-2-yl)-2,5,-diphenyltetrazolium bromide was obtained from VWR Life Science (Radnor, PA, USA). 2-Deoxy-[^3^H]-glucose was obtained from PerkinElmer Life Sciences (Boston, MA, USA). Rosiglitazone was obtained from Masung & Co., Ltd. (Seoul, Korea). All other chemicals were of analytical grade. Trans-Caffeic acid and syringaldehyde were purchased from Sigma-Aldrich (St. Louis, Mo, USA). Other standard compounds (morin 3-O-β-D-glucopyranoside, moracin M 3′-O-β-glucopyranoside, and astragalin) were provided by Professor Young Ho Kim in College of Pharmacy at Chungnam National University (Daejeon, South Korea). All standards were higher than 95% of purity. Methanol, ethyl acetate and acetonitrile were purchased with HPLC grade from Honeywell Burdick & Jackson (Muskegon, MI, USA). Formic acid (HPLC grade) was purchased from Sigma-Aldrich Corporation (St. Louis, MO, USA). Other chemicals used were GR (Guaranteed Reagent) grade. Ultrapure water was manufactured by a Milli-Q water purification system (ShinHan, Daejeon, South Korea). Anti-phospho-AKT serine 473 (S473) and AKT antibodies were obtained from Cell Signaling Technology, Inc. (Beverly, MA, USA). Anti-β-actin and anti-rabbit antibodies were purchased from AbFrontier (Geumcheon, Seoul, Korea).

### Mulberry leaf extraction

Dried mulberry leaves were purchased in September 2015 at the Naemome Dah herb company in Ulsan, Korea. The herb was taxonomically identified by Professor Young Ho Kim who is an expert in the area of Natural Product Chemistry. A voucher specimen (CNU 16004) was deposited at the Herbarium of College of Pharmacy, Chungnam National University, Daejeon, Korea. Dried leaves (100 g) were reconstituted in about 20 times its weight of purified water, pressure-extracted at 121 °C for 3 h, and then filtered. The filtered extract was concentrated using a vacuum concentrator (Rovator R-200; BÜCHI Korea Inc., Seoul, Korea) and 16 g powder was obtained using a freeze dryer. Solutions of the resulting MLE powder were created at a concentration of 300 or 600 mg/kg in normal saline for the animal experiments.

### Bioconversion of MLE with Viscozyme L

Water MLE was adjusted to pH 4.5, the enzyme Viscozyme L (Novozyme, Copenhagen, Denmark) was added at a concentration of 1% dried leaf, and the fermentation process was conducted at 45 °C for 15 h, after which the enzyme activity was terminated at 90 °C for 30 min. The solution was filtered, concentrated using a vacuum evaporator, and freeze-dried to obtain 19 g powder. The resulting BMLE powder was used at concentrations of 300 or 600 mg/kg in normal saline for the animal experiments.

### Sample preparation and HPLC analysis

The marker compounds in BMLE and MLE used in this study were analyzed by Prof. Jong Seong Kang in College of Pharmacy at Chungnam National University (Daejeon, South Korea). This method is under review for Analytical Journal.

### Animals

Five-week-old C57BL/6 mice (18–23 g) were purchased from OrientBio (Seongnam, Korea). The mice were acclimatized for 1 week at 22 ± 2 °C and 40 ± 5% humidity, and allowed free access to a normal- or high-fat diet (60% kcal fat, Research Diets, Inc., New Brunswick, NJ, USA) and drinking water. All animal handling was performed according to the guidelines of the Committee for Ethical Usage of Experimental Animals at Chungnam National University (CNU-00773; Daejeon, South Korea). In all experiments using animals, an inhalational anesthetic, isoflurane, was used to alleviate animal pain, and they were euthanized by cervical dislocation after completing experiments.

### Induction of obese diabetic mice

After fasting for 16 h, mice fed on high-fat diet for 8 weeks were intraperitoneally (IP) injected by NA (240 mg/kg) 15 min before administration of STZ (100 mg/kg, IP) to induce obese diabetic condition as described previously [28, 29, 33]. To confirm whether a diabetic condition was established in animals, oral glucose tolerance test (OGTT) was performed and the results were compared with type 1 diabetic mice induced by administration of STZ (100 mg/kg) alone. These results showed that STZ-NA-induced diabetic mice had different conditions from STZ-induced ones (Additional file 1).

### Experimental design

The experimental mice were randomly divided into eight groups containing seven mice in each group as follows:

- Group 1 (ND): normal-diet-fed mice treated with normal saline
- Group 2 (HFD): high-fat-diet-fed mice treated with normal saline
- Group 3 (Vehicle): obese diabetic mice treated with normal saline
- Group 4 (MLE-300 mg/kg): obese diabetic mice treated with 300 mg/kg MLE
- Group 5 (MLE-600 mg/kg): obese diabetic mice treated with 600 mg/kg MLE
- Group 6 (BMLE-300 mg/kg): obese diabetic mice treated with 300 mg/kg BMLE
- Group 7 (BMLE-600 mg/kg): obese diabetic mice treated with 600 mg/kg BMLE
- Group 8 (Metformin-300 mg/kg): obese diabetic mice treated with 300 mg/kg metformin as a positive control. All agents or vehicles were daily oral-administered to animals in all groups for 7 weeks.

### Oral glucose tolerance test

One week before the end of the experiment, all mice in the eight groups were fasted for 16 h. Glucose (2 g/kg, PO) was administered 30 min after the administration of the vehicle or agents, and blood was collected from the tail vein of each animal at 0, 15, 30, 45, 60, and 120 min to measure blood glucose with a blood glucose meter (Accu-Chek; Roche Diagnostics Korea Co., Ltd., Seoul, Korea). The OGTT data were plotted using GraphPad, and significant differences among groups were evaluated by measuring the AUC of each line graph.

### Insulin tolerance test

The ITT followed the same protocol as the OGTT, except for the subcutaneous administration of insulin (1 U/kg) 30 min after glucose administration (2 g/kg, PO). Blood was collected from the tail vein of each animal after 0, 30, 45, 60, and 120 min, and blood glucose was measured. The data interpretation was the same as that described for the OGTT.

### Insulin measurement

Mice were fasted for 16 h before the final administration of vehicle or agent. Mice were anesthetized 30 min after the final administration via isoflurane inhalation (2–3% isoflurane with oxygen supply; Isoflurane 100, JW PHARMA, Daejeon, Korea). After laparotomy, about 0.7–1 mL blood was collected through the abdominal artery in the EDTA-coated tube. Plasma was separated by centrifugation at 4000 rpm for 15 min, and plasma insulin was measured with a mouse insulin ELISA kit (Cat. AKRIN-011 T; Shivayagi, Gunma, Japan) according to the manufacturer’s instructions. The amount of insulin released from HIT-T15 cells into the media was measured as previously described [34]. HIT-T15 pancreatic β-cells seeded on a 24-well at a density of 2 × 10^5^ cells/well were treated with RPMI-1640 medium supplemented with 10% FBS and 11.1 mM glucose and were cultured for 2 days. After washing with Krebs-Ringer bicarbonate-HEPES (KRBH) buffer (119 mM NaCl, 4.74 mM KCl, 2.54 mM CaCl_2_·2H_2_O, 1.19 mM MgCl_2_·6H_2_O, 1.19 mM KH_2_PO_4_, 25 mM NaHCO_3_, and 7 mM HEPES; pH 7.4) without glucose, cells were incubated in KRBH buffer with 0.3% bovine serum albumin for 30 min, which was exchanged with KRBH buffer containing 11.1 mM glucose, and then treated with the prescribed agent for 1 h. Insulin levels released from cells into the KRBH buffer were measured with an insulin assay kit as described above. After lysing cells with 150 μL 0.5 N NaOH, the protein concentration in cell lysate was measured. Standard curves were established using the standard materials included in the ELISA kit, and plasma insulin levels (ng/mL) and insulin secretion (ng insulin/mg protein) were measured using a microplate reader (Infinite M200 PRO; Tecan Group Ltd., Zürich, Switzerland) at 450 nm.

### HbA_1C_ analyses

HbA_1C_ levels in the whole blood of mice were measured using a mouse HbA_1C_ Assay Kit (Cat. 80,310; Crystal Chem, Elk Grove Village, IL, USA) according to the manufacturer’s instructions. After establishing a standard curve, HbA_1C_ levels were determined using a microplate reader at 700 nm [35].

### Calculation of HOMA-IR and QUICKI

The HOMA-IR and QUICKI indices were calculated as follows:

$$ \mathrm{HOMA}-\mathrm{IR}=\frac{fasting\ insulin\ \left(\raisebox{1ex}{$\mu U$}\!\left/ \!\raisebox{-1ex}{$ mL$}\right.\right)\times fasting\ glucose\ \left(\raisebox{1ex}{$ mg$}\!\left/ \!\raisebox{-1ex}{$ dL$}\right.\right)}{405} $$

$$ \mathrm{QUICKI}=\frac{1}{\left[\log fasting\ insulin\ \left(\raisebox{1ex}{$ mU$}\!\left/ \!\raisebox{-1ex}{$L$}\right.\right)+\log fasting\ glucose\ \left(\raisebox{1ex}{$ mg$}\!\left/ \!\raisebox{-1ex}{$ dL$}\right.\right)\right]} $$

### Cell culture and differentiation

C2C12 myotubes and 3 T3-L1 adipocytes were cultured and differentiated as previously described [36, 37]. Briefly, C2C12 myoblasts were cultured at 37 °C under a 5% CO_2_ atmosphere in DMEM containing 10% FBS. Confluent myoblasts were designated as day 0, and DMEM containing 1% FBS was replaced daily for 6 days to differentiate C2C12 myotubes. 3 T3-L1 preadipocytes were cultured at 37 °C in a 5% CO_2_ atmosphere in DMEM containing 10% calf serum for 2 days, and confluent 3 T3-L1 cells were designated as day 0. Cells at day 0 were treated with 10% FBS, 0.5 mM IBMX, 1 μM dexamethasone, and 1 μg/mL insulin for 3 days to induce differentiation. After 3 days of differentiation, cells were maintained in DMEM containing 10% FBS and 1 μg/mL insulin for 2 days. Then the cell medium was replaced with DMEM containing 10% FBS, which was replaced every 2 days until day 8. HIT-T15 pancreatic β-cells were cultured at 37 °C in a 5% CO_2_ atmosphere in RPMI-1640 culture medium containing 10% FBS, 0.8 mM glucose, 100 U/mL penicillin, and 100 μg/mL streptomycin, and were subcultured for 3–5 days [34].

### Glucose uptake measurement

3 T3-L1 adipocytes or C2C12 myotubes grown in DMEM with 1% FBS were treated with Krebs-Ringer phosphate-HEPES buffer (136 mM NaCl, 4.7 mM KCl, 1 mM MgSO_4_, 1 mM CaCl_2_, 10 mM phosphate buffer, and 10 mM HEPES; pH 7.4). Cells were treated with medium alone (basic glucose uptake) or with 100 nM insulin (insulin-stimulated glucose uptake) for 30 min. Glucose uptake was initiated with adding 100 μM 2-deoxy-D-glucose together with 2-deoxy-[^3^H]-glucose (0.1 μCi/mL in 3 T3-L1 adipocytes or 0.5 μCi/mL in C2C12 myotubes) per well. After 10 min, the cells were washed three times with cold PBS and lysed using 0.5 M NaOH and 0.1% sodium dodecyl sulfate. The radioactivity was determined using liquid scintillation counting with a β-counter (1600TR; BioSurplus, Inc., San Diego, CA, USA) and normalized according to the total protein level [36, 37].

### Western blot analysis

Total proteins in differentiated C2C12 cells were extracted using RIPA buffer and quantified with BCA protein assay kit (Thermo Fisher Scientific, Rockford, IL, USA). Proteins in the sample buffer were boiled for 10 min and resolved by 10% sodium dodecyl sulfate polyacrylamide gel electrophoresis (SDS-PAGE). Proteins were transferred to a polyvinylidene fluoride (PVDF) membrane (ATTO Corp., Tokyo, Japan) at 4 °C for 80 min at 120 mA. After blocking with TBS-T (10 mM Tris, 150 mM NaCl, 0.1% Tween-20, pH 7.6) containing 5% bovine serum albumin (BSA) for 1 h, the membranes were then incubated with a 1:1000 dilution of primary antibodies targeting the following; phospho-AKT (S473), AKT, and β-actin. After removing primary antibodies, the blots were washed three times in TBS-T, incubated with anti-rabbit antibody diluted 1:2000 in TBS containing 5% BSA for 5 h at 4 °C and then washed five times in TBS-T. Antibody-bound proteins were detected using enhanced chemiluminescence (AbFrontier, Seoul, Korea) and imaging systems (Bio-Rad, Hercules, CA, USA) according to the manufacturer’s instruction. The expression level of each protein was normalized to β-actin and the intensities of the bands were quantified using Quantity One (Bio-Rad, Hercules, CA, USA) [38, 39].

### Statistical processing

We statistically analyzed the experimental results with Dunnett’s test after performing one-way analysis of variance. All data are expressed as the means ± standard error of the mean (SEM), and *p* < 0.05 was considered statistically significant.

## Results

### The content of two marker compounds and their abilities to increase glucose uptak in C2C12 myotubes

**Fig. 1:**
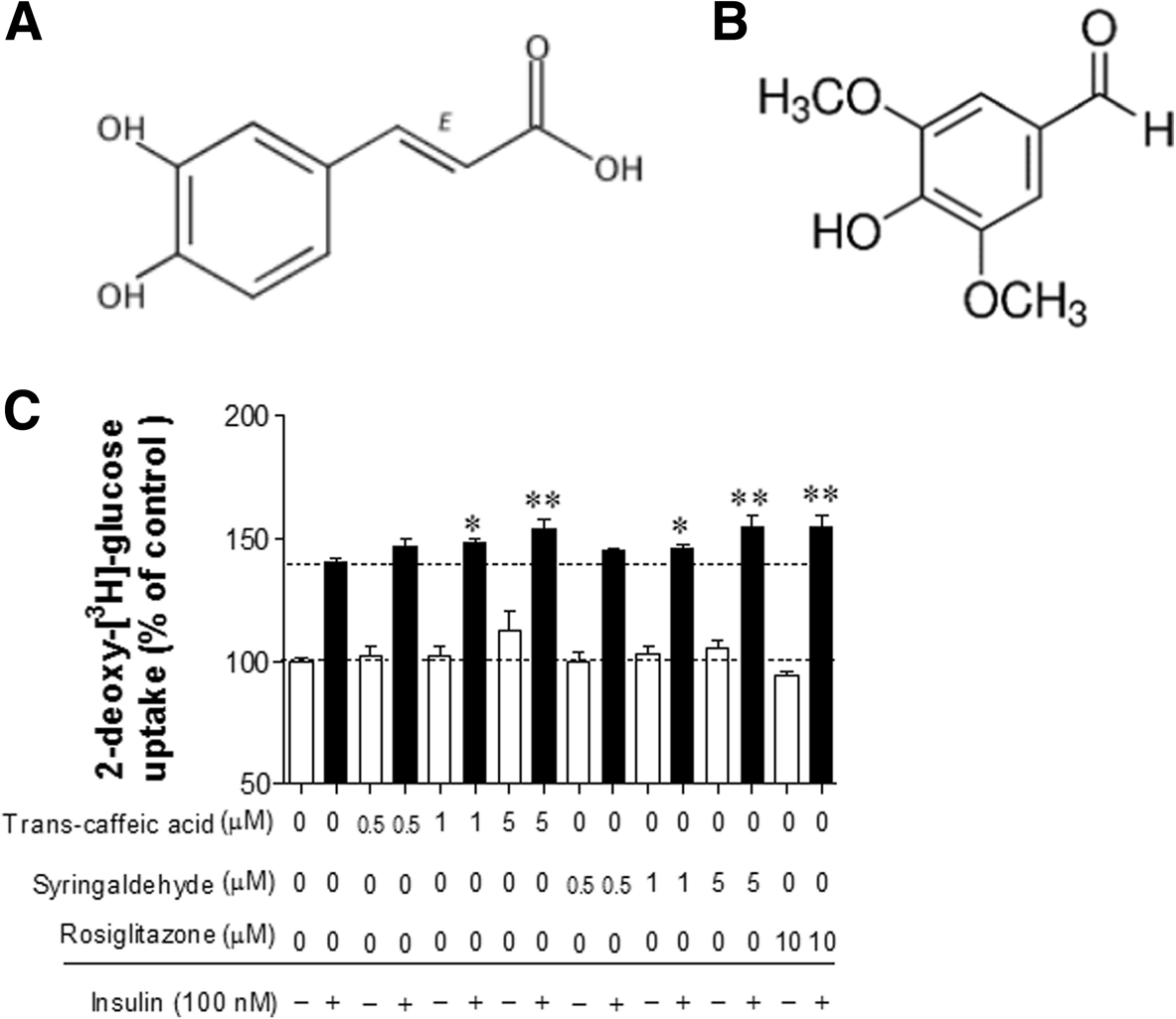
Structures of two marker compounds in mulberry leaf extract and the effect of these two compounds on glucose uptake in differentiated C2C12 myotubes (**c**). Structures of trans-caffeic acid (**a**) and syringaldehyde (**b**). Differentiated C2C12 cells were incubated in serum-starved DMEM for 1 h and then treated with trans-caffeic acid or syringaldehyde 1 h. Thereafter, the medium was changed to Krebs-Ringer phosphate-HEPES buffer containing the prescribed concentrations of trans-caffeic acid or syringaldehyde for 30 min, and then cells were treated with 100 nM insulin for 30 min. After treatment with 2-deoxy-[^3^H]–glucose (0.5 μCi/mL) and 2-deoxy-D-glucose (100 μM) for 10 min, the reaction was terminated and glucose uptake activity was measured in lysed cells with a liquid scintillation counter (see Materials and methods). Rosiglitazone (10 μM) was used as a positive control for glucose uptake in differentiated C2C12 myotubes. Data are expressed as the mean ± SEM of three independent experiments. * *P* < 0.05 and ** *P* < 0.01 versus insulin-stimulated glucose uptake in the untreated control

**Fig. 2:**
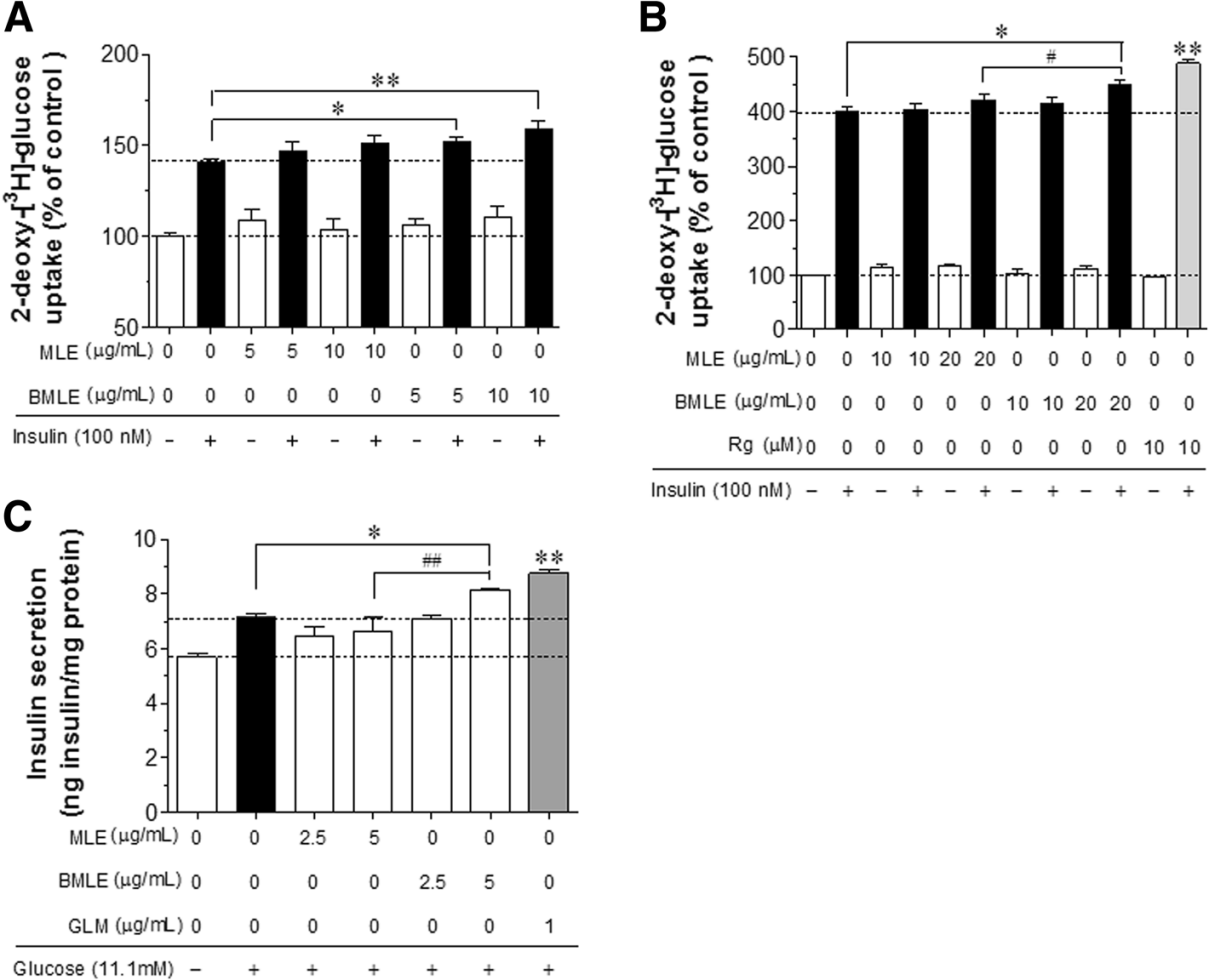
Comparison of the abilities of bioconverted mulberry leaf extract (BMLE) and unaltered mulberry leaf extract (MLE) to improve glucose uptake and insulin secretion in (**a**) differentiated C2C12 myotubes, (**b**) 3 T3-L1 adipocytes, and (**c**) HIT-T15 pancreatic β-cells. To measure glucose uptake, cells were incubated in serum-starved DMEM for 1 h and then, treated with MLE, BMLE or rosiglitazone (Rg) for 1 h. The medium was changed to Krebs-Ringer phosphate-HEPES buffer containing the prescribed concentrations of MLE, BMLE or Rg for 30 min and then treated with 100 nM insulin for 30 min. After treatment with 2-deoxy-[^3^H]–glucose (0.5 μCi/mL) and 2-deoxy-D-glucose (100 μM) for 10 min, the reaction was terminated and glucose uptake activity was measured in lysed cells with a liquid scintillation counter (see Materials and methods). To measure insulin secretion, cells incubated with Krebs-Ringer bicarbonate-HEPES buffer containing 0.3% bovine serum albumin for 30 min were treated with 11.1 mM glucose and MLE, BMLE or glimepiride (GLM) for 1 h and the insulin concentration released into the media was measured (see Materials and Methods). Rg (10 μM) and GLM (1 μg/mL) were used as positive controls for glucose uptake in differentiated C2C12 myotubes and 3 T3-L1 adipocytes and insulin release in HIT-T15 pancreatic β-cells, respectively. Data are expressed as the mean ± SEM of three independent experiments. * *P* < 0.05 and ** *P* < 0.01 versus insulin-stimulated glucose uptake or glucose-stimulated insulin release in the untreated control; ^#^ *P* < 0.05 and ^##^ *P* < 0.01 versus insulin-stimulated glucose uptake or glucose-stimulated insulin release in the MLE-treated group

HPLC analysis showed two major marker compounds in MLE, compounds **1** (trans-caffeic acid, Fig. 1a) and **2** (syringaldehyde, Fig. 1b), and the quantities of these two compounds in BMLE were increased by 184 and 280%, respectively (Table 1), indicating that bioconversion process increases the quantity of marker compounds compared with unaltered MLE. To confirm the abilities of two marker compounds to uptake glucose into cells, two compounds with variable concentrations (0.5–5 μM) were applied to C2C12 myotubes. Both trans-caffeic acid and syringaldehyde significantly increased insulin-stimulated glucose uptake in a concentration-dependent manner (Fig. 1c). These results indicate that the more concentration of these compounds provides the greater ability of glucose uptake.

**Table 1:** Quantitative analysis of two marker compounds in mulberry leaf extract (MLE) and bioconverted mulberry leaf extract (BMLE). (*n* = 6)

Compound No	MLE	BLME	BLME/MLE
	Conc. (mg/g)^a^	Conc. (mg/g)^a^	
trans-caffeic acid	0.393 ± 0.024	0.725 ± 0.012	1.84
syringaldehyde	0.206 ± 0.009	0.577 ± 0.016	2.80

^a^Data are represented as average ± SD

### Comparison of the abilities of BMLE and MLE to increase glucose uptake and insulin secretion

We compared the abilities of BMLE and MLE to increase glucose uptake in differentiated C2C12 myotubes and 3 T3-L1 adipocytes and insulin secretion from HIT-T15 pancreatic β-cells. Insulin-stimulated glucose uptake significantly increased in C2C12 myotubes treated with 5 or 10 μg/mL BMLE (Fig. 2a). BMLE treatment increased insulin-stimulated glucose uptake from 140 to 152% at a concentration of 5 μg/mL and to 159.1% at 10 μg/mL. By contrast, MLE showed no significant increase in glucose uptake. Neither MLE nor BMLE affected basal glucose uptake. Similarly, 20 μg/mL BMLE significantly increased insulin-stimulated glucose uptake in 3 T3-L1 adipocytes (Fig. 2b). Moreover, the ability of BMLE (20 μg/mL) to increase insulin-stimulated glucose uptake (from 400 to 449.3%) was significantly greater than that of MLE (20 μg/mL; from 400 to 420.7%). As a positive control, rosiglitazone (10 μM) significantly increased insulin-stimulated glucose uptake up to 487.9%. Although 20 μg/mL BMLE significantly increased insulin secretion in HIT-T15 pancreatic β-cells, MLE did not show any stimulating effect (Fig. 2c). As a positive control, glimepiride (1 μg/mL) showed the greatest efficacy in stimulating insulin secretion in this model. These results indicate that bioconversion improved the ability of MLE to increase insulin-stimulated glucose uptake in skeletal muscle cells and adipocytes, as well as insulin secretion in pancreatic β-cells. BMLE and MLE at greater doses (20 or 50 μg/mL) than those used in the experiments showed no cytotoxicity for 1 or 2 h in these cells (In vitro test of the cytotoxicity; Additional file 2).

### The effect of BMLE, MLE and two marker compounds on AKT phosphorylation in C2C12 myotubes

**Fig. 3:**
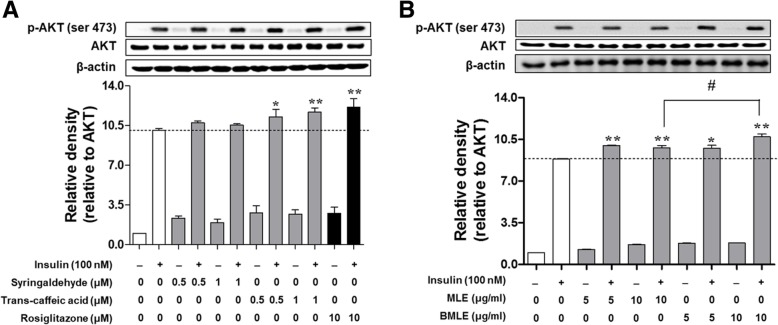
Effect of two marker compounds (**a**) and unaltered mulberry leaf extract (MLE) and bioconverted mulberry leaf extract (BMLE) (**b**) on AKT phosphorylation in insulin-stimulated C2C12 myotubes. Cells were incubated in serum-starved DMEM for 1 h and then treated with two marker compounds, MLE, BMLE, or rosiglitazone as a positive control for 1 h. The medium was changed to Krebs-Ringer phosphate-HEPES buffer containing two marker compounds, MLE, BMLE or rosiglitazone for 30 min. Thereafter, 100 nM insulin was treated for 30 min. Cells were lysed and proteins were employed by SDS-PAGE followed by Western blotting using primary antibodies targeting anti-phospho AKT, AKT and β-actin. The data depicted in bar graph expressed as the means ± SEM are an average of three similar and independent experiments and gel images are of representative blots. * *P* < 0.05 and ** *P* < 0.01 versus insulin-stimulated cells with no treatment; ^#^ *P* < 0.05 versus insulin-stimulated cells treated with MLE

Insulin-mediated AKT phosphorylation induces GLUT4 translocation from cytosol to membrane, enhancing glucose uptake [40, 41]. To confirm this linkage, the effect of two marker compounds, MLE and BMLE on AKT phosphorylation was measured in C2C12 myotubes (Fig. 3). Trans-caffeic acid significantly increased AKT phosphorylation (Fig. 3a). Syringaldehyde did not show any statistical significant increase at given concentrations, but exert an increasing tendency. As expected, both BMLE and MLE significantly increased AKT phosphorylation (Fig. 3b). Notably, BMLE had a greater effect on AKT phosphorylation than MLE at the same concentration (10 μg/mL). These results suggest that bioconversion increase in amount of trans-caffeic acid, which causes the greater effect than MLE on AKT phosphorylation-induced glucose uptake.

### Improvement of glucose tolerance in obese diabetic mice by BMLE and MLE treatment

**Fig. 4:**
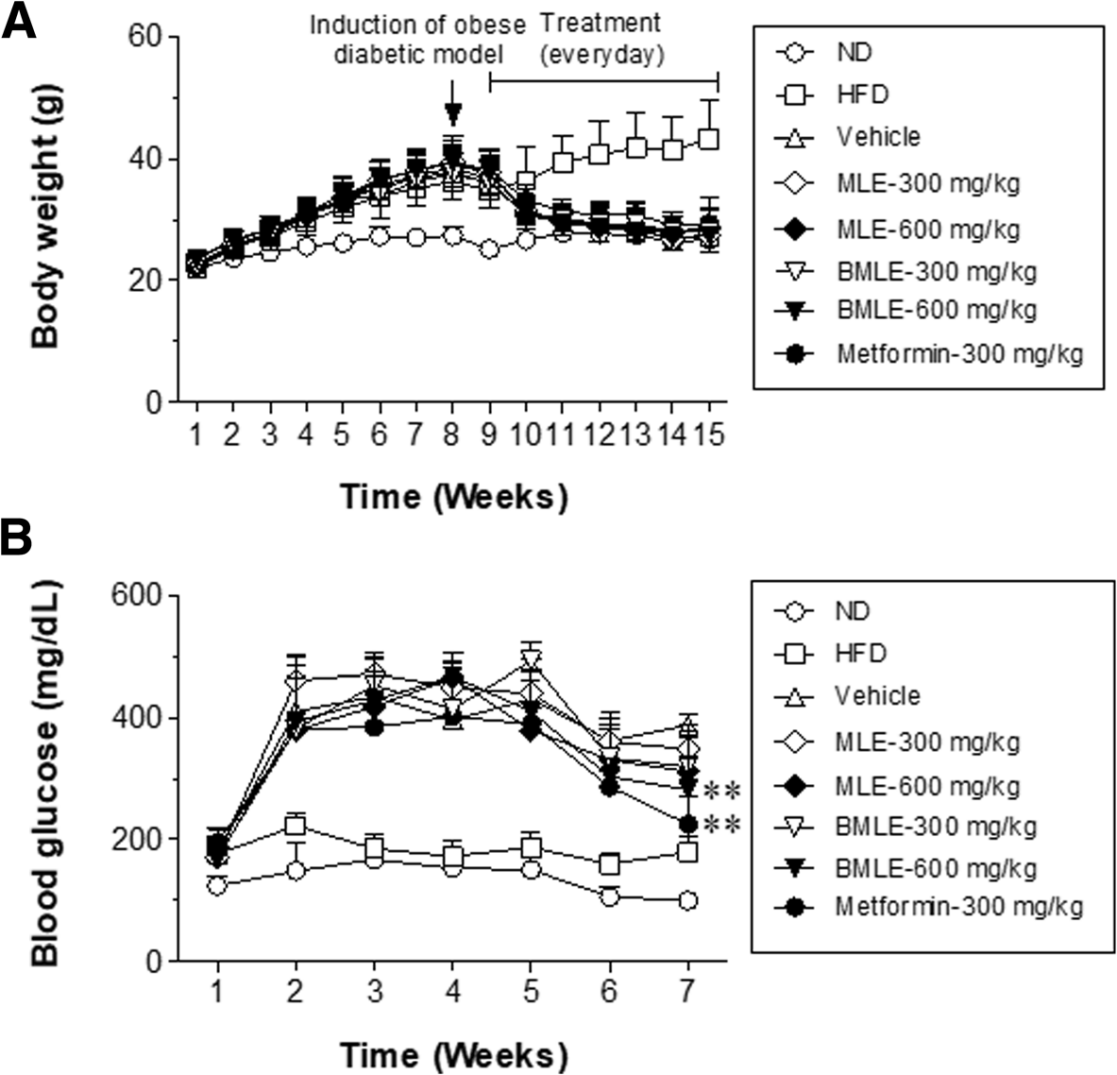
Effects of bioconverted mulberry leaf extract (BMLE) compared to unaltered mulberry leaf extract (MLE) on the body weight and blood glucose levels of obese diabetic mice. Obese diabetic mice were produced by feeding on high-fat diet (HFD) for 8 weeks into six-week-old C57BL/6 mice and then (at the beginning of 9th week) injecting streptozotocin with nicotinamide (see Materials and methods). After checking for high blood glucose, mice were orally administered with MLE (300 and 600 mg/kg) or BMLE (300 and 600 mg/kg) for 7 weeks and the changes in (**a**) body weight and (**b**) blood glucose level were examined. Metformin (300 mg/kg) was used as a positive control. Data are expressed as the mean ± SEM (*n* = 7). ** *P* < 0.01 versus blood glucose levels in vehicle-control mice

To compare the abilities of BMLE and MLE to improve glucose tolerance, obese diabetic mice were established by co-administering STZ and NA as described previously [28, 29]. The body weights of both BMLE- and MLE-treated mice fed a high-fat diet decreased (Fig. 4a). As a positive control, metformin (300 mg/kg, PO) treatment also resulted in a decrease in body weight. However, fasting blood glucose levels measured once a week were significantly decreased in both the BMLE (600 mg/kg, PO)- and metformin-treated groups (Fig. 4b). Note that the quantities of two marker compounds in BMLE dosage is greater than those in MLE dosage (Table 2).

**Table 2:** The total quantities of two marker compounds in injected doses of mulberry leaf extract (MLE) and bioconverted mulberry leaf extract (BMLE) in animal experiments

Compounds	Dosage	MLE (mg)	BLME (mg)
trans-caffeic acid	300 mg/kg	0.1179	0.2175
	600 mg/kg	0.2358	0.435
syringaldehyde	300 mg/kg	0.0618	0.1236
	600 mg/kg	0.1236	0.3462

**Fig. 5:**
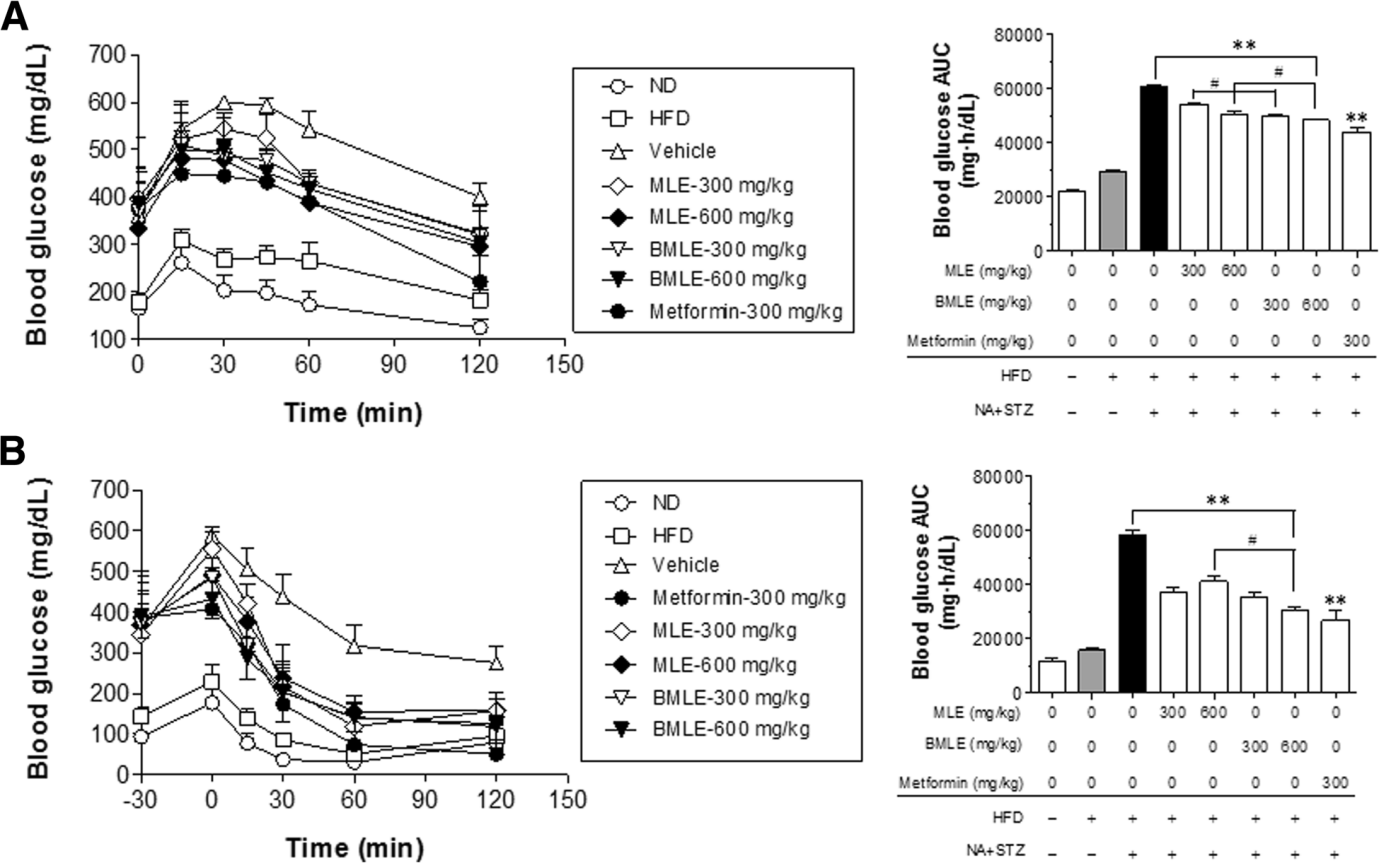
Effects of bioconverted mulberry leaf extract (BMLE) and unaltered mulberry leaf extract (MLE) on oral glucose tolerance test (OGTT) and insulin tolerance test (ITT) in obese diabetic mice. MLE (300 and 600 mg/kg) or BMLE (300 and 600 mg/kg) were administered (PO) for 7 weeks in obese diabetic mice. Blood was collected from the tail vein (**a**) 15, 30, 45, 60, and 120 min after oral administration of glucose (2 g/kg) for the OGTT and (**b**) 30, 45, 60, and 120 min after administration (SC) of insulin (1 U/kg) for the ITT (see Materials and Methods). Metformin (300 mg/kg) was used as a positive control. Data are expressed as the mean ± SEM (n = 7), and significant changes among experimental groups were interpreted by converting all data into the area under the curve (AUC). ** *P* < 0.01, versus high-fat diet-fed (HFD) diabetic mice control (vehicle treatment), and ^#^ *P* < 0.05, vs. MLE

The oral glucose tolerance test (OGTT) was performed after 6 weeks of treatment, and the results were plotted with a line graph, using the area under the curve (AUC) of each line to compare the differences among treatments (Fig. 5a). Both the BMLE- and MLE-treated groups showed significantly decreased blood glucose levels compared to the control (untreated obese diabetic mice fed a high-fat diet). The blood glucose levels in the BMLE-treated group were lower than those in the MLE-treated group at the same dose. The insulin tolerance test (ITT) was performed after 7 weeks of treatment, and significant changes among groups were compared based on the AUC (Fig. 5b). After injecting insulin, blood glucose levels in both the BMLE- and MLE-treated groups were significantly lower than those in the control group. Moreover, the AUC was significantly lower in the 600 mg/kg BMLE-treated group than in the 600 mg/kg MLE-treated group. In both tests, the AUCs of metformin were similar to those of the high concentration-treated BMLE group. These results indicate that BMLE had a better ability to regulate blood glucose levels in the obese diabetic animal model than MLE.

### Regulation of HbA_1C_ levels and insulin plasma levels by BLME and MLE

**Fig. 6:**
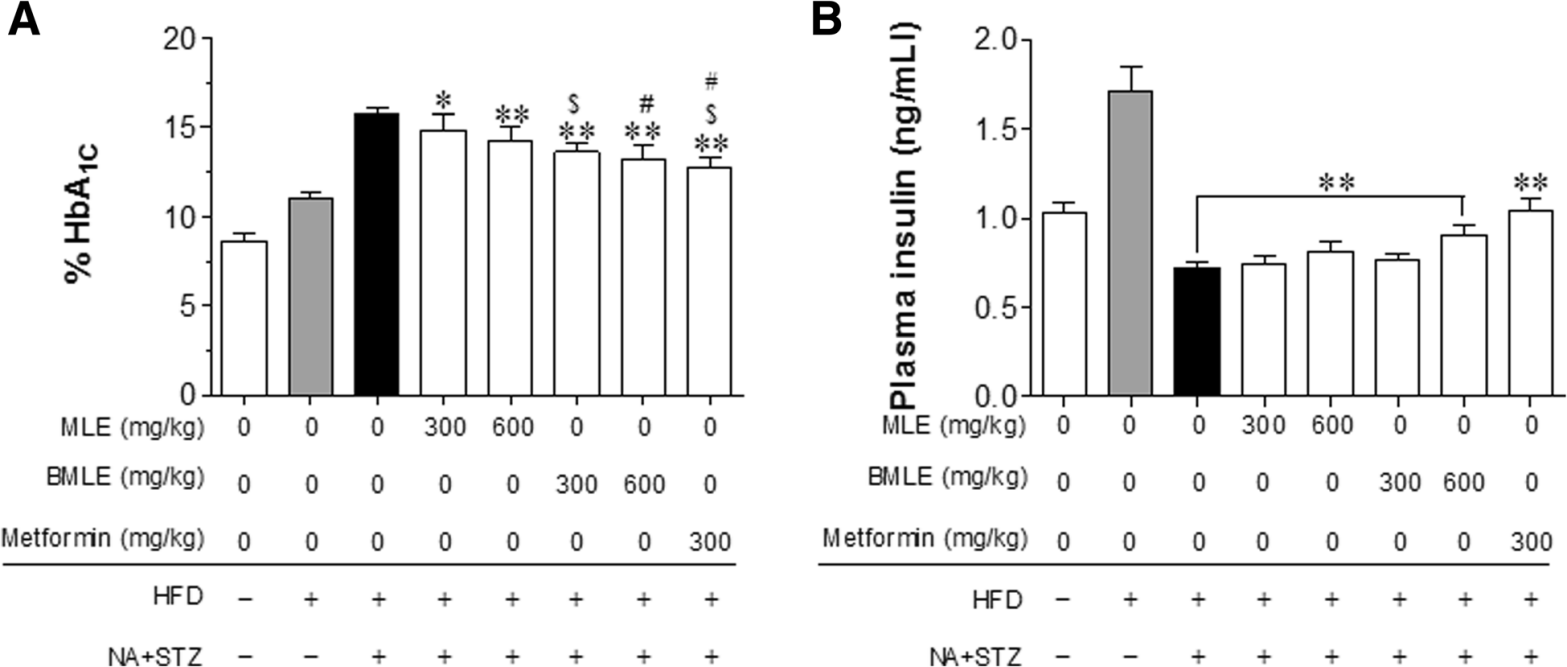
Effect of bioconverted mulberry leaf extract (BMLE) and unaltered mulberry leaf extract (MLE) on HbA_1C_ blood concentrations and plasma insulin levels in obese diabetic mice. MLE (300 and 600 mg/kg) or BMLE (300 and 600 mg/kg) were administered (PO) for 7 weeks in obese diabetic mice, and (**a**) the amount of glycated hemoglobin (HbA_1C_) in blood and (**b**) plasma insulin level was measured. Data are expressed as the mean ± SEM (*n* = 7). * *P* < 0.05 and ** *P* < 0.01 versus the vehicle; ^$^ *P* < 0.05 versus the MLE-treated group (300 mg/kg); ^#^ *P* < 0.05 versus the MLE-treated group (600 mg/kg). HFD, high-fat diet; NA, nicotinamide; STZ, streptozotocin

**Fig. 7:**
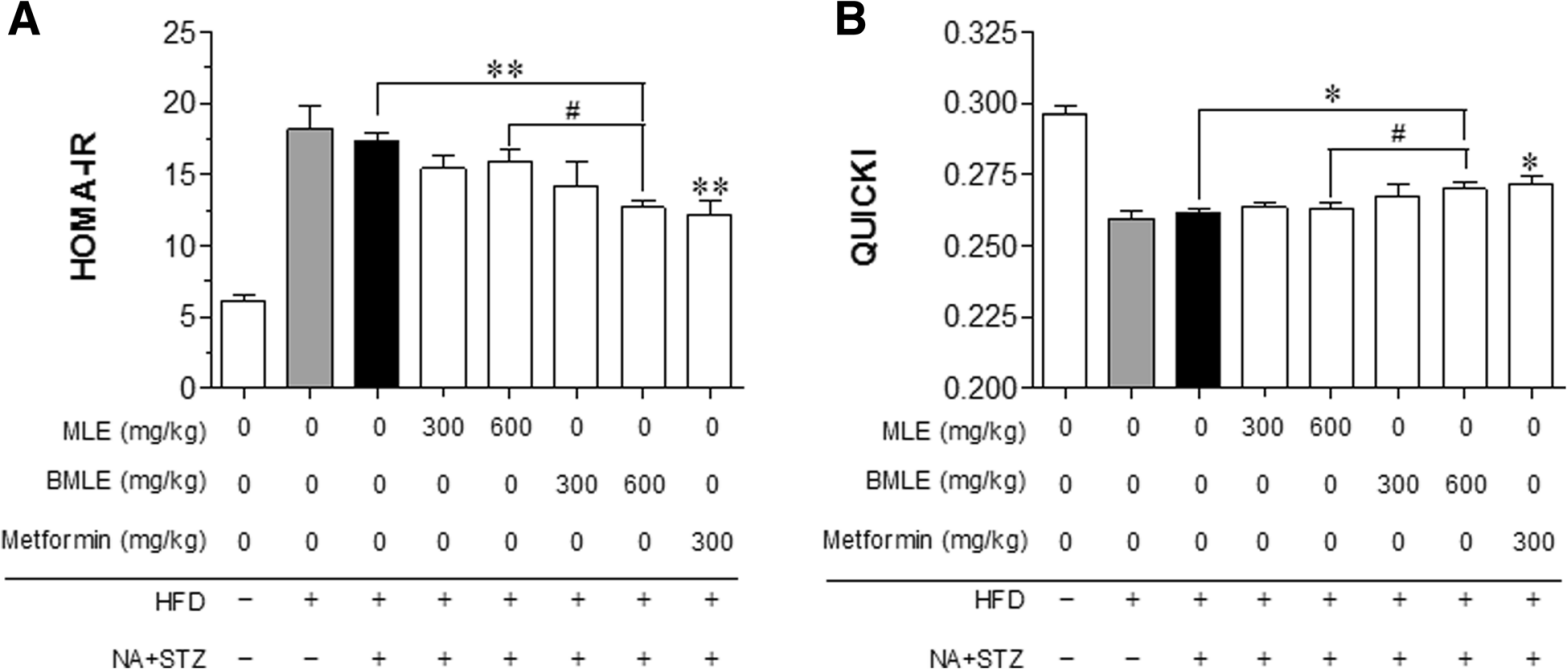
Effects of bioconverted mulberry leaf extract (BMLE) and unaltered mulberry leaf extract (MLE) on indices of insulin resistance and insulin sensitivity in obese diabetic mice. MLE (300 and 600 mg/kg) or BMLE (300 and 600 mg/kg) were administered (PO) for 7 weeks in obese diabetic mice, and (**a**) homeostasis model assessment of insulin resistance (HOMA-IR) and (**b**) quantitative insulin sensitivity check index (QUICKI) values were calculated by measuring fasting glucose and insulin levels. Data are expressed as the mean ± SEM (*n* = 7). * *P* < 0.05 and ** *P* < 0.01 versus the vehicle; ^#^ *P* < 0.05 versus the MLE-treated group (600 mg/kg). HFD, high-fat diet; NA, nicotinamide; STZ, streptozotocin

After the final BMLE and MLE treatment in mice, HbA_1C_ blood and plasma insulin levels were measured. The HbA_1C_ levels in both the BMLE- and MLE-treated groups were significantly lower than those in the control group (Fig. 6a). As expected, the HbA_1C_ levels in the BMLE-treated group were significantly lower than those of the MLE-treated group at the same dosage. Plasma insulin levels were significantly increased in only the group treated with 600 mg/kg BMLE (Fig. 6b). Next, the homeostasis model assessment of insulin resistance (HOMA-IR) and quantitative insulin sensitivity check index (QUICKI) values were compared among the groups (for the calculations, see the Materials and Methods). The HOMA-IR value in the 600 mg/kg BMLE-treated group was significantly lower than that in the MLE group, but was similar to that in the metformin-treated group (Fig. 7a). Moreover, the QUICKI value of the 600 mg/kg BMLE-treated group was higher than that of the MLE group (Fig. 7b). These results indicate that BMLE showed a better capacity than MLE to decrease blood HbA_1C_ levels, increase insulin secretion, and improve insulin resistance and insulin sensitivity.

## Discussion

We investigated the differences in the pharmacological efficacies of BMLE and MLE. Bioconversion process provides the more quantities of two marker compounds, trans-caffeic acid and syringaldehyde, and these two compounds have an ability to increase glucose uptake in C2C12 myotubes in a dose-dependent manner. Syringaldehyde has been known to increase glucose uptake in rat L6 myocytes [42] and GLUT-4 expression in skeletal muscles isolated from STZ-induced diabetic rats [43]. Previous reports showed that caffeic acid enhanced glucose uptake in tumor necrosis factor α-treated insulin-resistant FL83B cells [44] and human skeletal muscle cells (SkMC C-12580) [45], and increased insulin secretion in rat insulinoma cells (INS-1E) [45]. At the cellular level, BMLE was more effective than MLE in increasing glucose uptake into skeletal muscle cells and adipocytes, increasing insulin secretion in pancreatic β-cells and lowering blood glucose. In a diabetic animal model, BMLE more effectively controlled fasting blood glucose and insulin levels than MLE. Therefore, our results demonstrate that bioconversion can increase the pharmacological efficacy of MLE, which may be due to the greater concentration of two marker compounds in BMLE.

Previous reports have shown that MLE stimulates glucose uptake in rat adipocytes [46] and significantly lowers fasting plasma glucose and increases insulin secretion in STZ-induced diabetic rats [47]. Consistent with previous reports, our observations (Fig. 2a, b) revealed that both BMLE and MLE increased glucose uptake into C2C12 myotubes and 3 T3-L1 adipocytes; however, BMLE exhibited greater pharmacological efficacy than MLE. Moreover, the ability of BMLE to increase insulin secretion from pancreatic β-cells was greater than that of MLE (Fig. 2c). Moreover, both BMLE and MLE have an ability to significantly increase AKT phosphorylation in C2C12 myotubes (Fig. 3). Since BMLE has a greater effect on AKT phosphorylation than MLE at the same concentration, BMLE is superior to increase glucose uptake than MLE. These in vitro results indicate that BMLE is more effective for controlling blood glucose levels than MLE at the same concentration.

Normalization of blood glucose levels is important for the management of diabetes, and some herbal remedies are effective for improving hyperglycemia. MLE has been shown to reduce fasting blood glucose and blood HbA_1C_ levels in STZ-induced diabetic rats by attenuating non-esterified fatty acid (NEFA) signaling and modulating intestinal microflora [13]. In this study using a diabetic animal model induced with a previously described method [28], BMLE significantly reduced fasting blood glucose levels (Fig. 4b), while effectively controlling blood glucose after administration of high glucose (Fig. 5a) and normalizing insulin function under high-glucose conditions (Fig. 5b). Compared to MLE, BMLE yielded greater effects at the same concentration. Moreover, both BMLE and MLE treatment significantly reduced blood levels of HbA_1C_, which were formed under hyperglycemic conditions [48], although the ability of BMLE to decrease HbA_1C_ blood levels was greater than that of MLE (Fig. 6a). Because type 2 diabetes causes the destruction of pancreatic β-cells via oxidative stress and decreases insulin secretion [49], the ability of high-fat-diet-fed diabetic mice to secret insulin was reduced, and the high concentration of BMLE (600 mg/kg) significantly restored insulin secretion (Fig. 6b). Taken together, BMLE can more effectively regulate blood glucose levels and restore insulin secretion in diabetic mice, leading to a decrease in HbA_1C_ levels in blood.

Improvements in insulin resistance [50] and insulin sensitivity [51] are the most important therapeutic parameter in patients with type 2 diabetes. HOMA-IR and QUICKI are commonly used as indicators of insulin resistance and β-cell function and of insulin sensitivity, respectively. Although both BMLE and MLE tended to improve the HOMA-IR and QUICKI indices, only the high concentration of BMLE showed a significant decrease in HOMA-IR (Fig. 7a) and increase in QUICKI (Fig. 7b) indices, which indicates that the high concentration of BMLE significantly improved insulin resistance and insulin sensitivity. Therefore, BMLE offers superior control of hyperglycemia and improvements in insulin resistance and insulin sensitivity than MLE, which suggests that bioconversion may be an important process for maximizing the pharmacological efficacy of natural product extracts.

## Conclusions

In this study, we examined the ability of BMLE to increase glucose uptake in C2C12 myotubes and 3 T3-L1 adipocytes, as well as insulin secretion in HIT-T15 pancreatic β-cells. Compared to MLE, treatment with BMLE in diabetic mice resulted in the improved ability to decrease fasting blood glucose levels, tolerate glucose, decrease blood HbA_1C_ levels, and increase plasma insulin, leading to the amelioration of insulin resistance and insulin sensitivity. Taken together, these results indicate that BMLE is superior to MLE in the management of diabetic condition, which suggests that bioconversion can be used as a useful tool for increasing the active ingredients in MLE.

## Additional files

Additional file 1: The following information is available online: Grouping experimental animals by results of blood glucose levels and oral glucose tolerance test. (PDF 1658 kb)

Additional file 2: The following information is available online: In vitro test of the cytotoxicity of the bioconverted and unaltered mulberry lead extracts (BMLE and MLE, respectively) used in this study. (PDF 1633 kb)

## Abbreviations

AUC: Area under the curve
BMLE: Bioconverted mulberry leaf extract
DMEM: Dulbecco’s modified eagle medium
FBS: Fetal bovine serum
HbA_1C_: Glycated hemoglobin
HFD: High-fat diet
HOMA-IR: Homeostasis model assessment of insulin resistance
ITT: Insulin tolerance test
KRBH: Krebs-ringer bicarbonate-HEPES
MLE: Mulberry leaf extract
NA: Nicotinamide
NEFA: Non-esterified fatty acid
OGTT: Oral glucose tolerance test
PBS: Phosphate-buffered saline
QUICKI: Quantitative insulin sensitivity check index
STZ: Streptozotocin
