# Supplementary figures and images for: Effects of unaltered and bioconverted mulberry leaf extracts on cellular glucose uptake and antidiabetic action in animals
Source: BMC Complement Altern Med. 2019 Mar 6;19:55. doi: 10.1186/s12906-019-2460-5 (PMC6404318; doi:10.1186/s12906-019-2460-5)

**A**

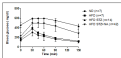

**B**

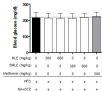

|  | control     |  | MLE         |             | DBLE        |             | Metformin |
|--|-------------|--|-------------|-------------|-------------|-------------|-----------|
|  |             |  | low         | high        | low         | high        |           |
|  | 217.3±34.26 |  | 214.2±31.36 | 212.7±29.85 | 212.2±29.18 | 217.3±26.80 | 226 ±26.9 |

Supplement: Supplementary file 1 — The following information is available online: Grouping experimental animals by results of blood glucose levels and oral glucose tolerance test. (PDF 1658 kb) [file 12906_2019_2460_MOESM1_ESM.pdf]

**A****C2C12 myotubes**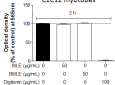**B****3T3-L1 adipocytes**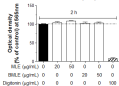**C****HIT-T15 pancreatic  $\beta$  cells**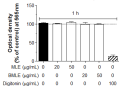

Supplement: Supplementary file 2 — The following information is available online: In vitro test of the cytotoxicity of the bioconverted and unaltered mulberry lead extracts (BMLE and MLE, respectively) used in this study. (PDF 1633 kb) [file 12906_2019_2460_MOESM2_ESM.pdf]
